# Review of Robotic Surgery in Gynecology—The Future Is Here

**DOI:** 10.5041/RMMJ.10296

**Published:** 2017-04-28

**Authors:** Roy Lauterbach, Emad Matanes, Lior Lowenstein

**Affiliations:** Department of Obstetrics and Gynecology, Rambam Health Care Campus, Haifa, Israel; and Ruth and Bruce Rappaport Faculty of Medicine, Technion–Israel Institute of Technology, Haifa, Israel

**Keywords:** Feasibility, high variance, robotic surgery, systematic review

## Abstract

The authors present a systematic review of randomized and observational, retrospective and prospective studies to compare between robotic surgery as opposed to laparoscopic, abdominal, and vaginal surgery for the treatment of both benign and malignant gynecologic indications. The comparison focuses on operative times, surgical outcomes, and surgical complications associated with the various surgical techniques. PubMed was the main search engine utilized in search of study data. The review included studies of various designs that included at least 25 women who had undergone robotic gynecologic surgery. Fifty-five studies (42 comparative and 13 non-comparative) met eligibility criteria. After careful analysis, we found that robotic surgery was consistently connected to shorter post-surgical hospitalization when compared to open surgery, a difference less significant when compared to laparoscopic surgery. Also, it seems that robotic surgery is highly feasible in gynecology. There are quite a few inconsistencies regarding operative times and estimated blood loss between the different approaches, though in the majority of studies estimated blood loss was lower in the robotic surgery group. The high variance in operative times resulted from the difference in surgeon’s experience. The decision whether robotic surgery should become mainstream in gynecological surgery or remain another surgical technique in the gynecological surgeon’s toolbox requires quite a few more randomized controlled clinical trials. In any case, in order to bring robotic surgery down to the front row of surgery, training surgeons is by far the most important goal for the next few years.

Robotic surgery used to sound like science fiction, the same as putting a man on the moon. With that said, as a matter of fact, some of the greatest advances in robotic surgery technology were inspired by NASA.

Robotic surgery was first used in 1983 in Canada, developed by an orthopedic surgeon and his team; the robot was named the “Arthrobot.” Since then, surgical robots evolved and were used in many fields of surgery from ophthalmology, through general surgery, urology, and gynecology. The “Arthrobot” was the first in a long line of surgical assisting robots, including some that handed surgical tools to the surgeon and others that performed surgery under the surgeon’s guidance or control. The next great step was in 1985. Kwoh and his associates performed a brain biopsy under CT guidance with the assistance of a robotic arm—PUMA560.^1^ Robotic surgery evolved through PROBOT (the first prostate surgical robot), ROBODOC (the first hip replacement surgical robot), and on to ZEUS—the da Vinci robot predecessor.

ZEUS was the first surgical robot to perform gynecological surgery when it was used in 1997 to reconnect fallopian tubes in state-of-the-art robotic surgery in Cleveland, Ohio, USA. By the end of the twentieth century, robotic surgery was used in thoracic surgeries for heart bypasses.

In the beginning of the twenty-first century, the first ever successful telerobotic procedure, a cholecystectomy, was performed utilizing the ZEUS robot. Surgery was performed by a surgeon based in New York on a patient operated on in France.^2^ The idea of telerobotics was even pitched to the Pentagon as a safe option that could enable surgical staff to treat injured soldiers on the battlefield without jeopardizing any more men. The project was named Mobile Advanced Surgical Hospital (MASH). Though never implemented at the time, the MASH system fueled the momentum for daily clinical implementation in the hospital environment.^3^

The company that developed the ZEUS robot was purchased by Intuitive Surgical, Inc., and after years of attempts to upgrade the system they brought out the da Vinci surgical robot. In 2005, the US Food and Drug Administration (FDA) approved the use of the da Vinci robotic system in gynecological surgery.

The year 2009 saw the publication of a large multi-institutional study on the use of the da Vinci robotic surgical system in gynecologic oncology, including learning curves for current and new users as a method to assess acquisition of their skills using the device.

Robotic surgery has evolved immensely over the last decade. Hundreds of studies have been published in the last 8 years, since the afore-mentioned study, in this field, ranging from theoretical to practical aspects.

A recent report by Intuitive Surgical, Inc. pointed out that during 2007–2013 the number of robotic surgical systems more than doubled in the US (from 800 to 2001) and Europe (from 200 to 443). The same report stated that 1.5 million robotic surgeries had been performed until 2013 in the world.

The da Vinci System was introduced in Israel in 2009. Currently, about 55% of all radical prostatectomies in Israel are robotic surgeries. At the time of writing, six robotic systems have been installed in six medical centers around Israel.

Surgeons at the Rambam Health Care Campus began robotic surgeries in November 2010 and in the field of gynecology in April 2011.

Robotic surgery in gynecology covers a broad spectrum of uses and is growing fast. The da Vinci Surgical System is used for benign indications such as treatment for fibroids, abnormal periods, endometriosis, pelvic prolapse, and ovarian growths, or for malignancies such as endometrial cancer, cervical cancer, or ovarian cancer. The robotic system may assist the gynecological surgeon in performing hysterectomies, salpingectomies, oophorectomies, myomectomies, and lymph node biopsies. Thus, abdominal surgery may become obsolete in the future—as we said, science fiction. Quite a few medical centers around the world have extensively studied the use of robotic surgery and found that it improves the morbidity and mortality rates of patients with gynecologic cancers.

The advantages for the patient may include shorter hospitalization, less pain and scarring, less risk of infection, less blood loss and fewer transfusions, faster recovery, and a quicker return to normal activities.

From a purely surgical point of view, the advantages of robotic surgeries over open and even laparoscopic surgeries are quite substantial and include:

1. A three-dimensional surgical view as opposed to the old laparoscopic two-dimensional view.
2. The ability to reduce to zero the effect of a surgeon’s tremor, adding to the precision and finesse of surgery.
3. The seven degrees of freedom of the robotic arms allow finer suturing and dissection of tissue with poor anatomic accessibility.
4. The ability to control three surgical instruments in addition to a camera enables the surgeon to operate with very little assistance, not having to rely on the assistant’s expertise.^4^

On the other hand, there are a few disadvantages of the robotic system, the most significant one being the high cost of equipment and maintenance.

In this review, we aim to summarize the latest studies and literature regarding robotic surgeries in the field of gynecology. Some studies portray state-of-the-art surgical techniques. Others report on a certain medical center’s or country’s robotic surgery experience. The rest may be the most important type of studies—the comparative studies, including open surgery versus robotic surgery, laparoscopic surgery versus robotic surgery, and different robotic surgeries.

## STUDY SOURCES AND SELECTION

We performed a systematic search to identify studies regarding robotic gynecologic surgery. PubMed was searched from inception to January 2017 for English-language studies, using the search terms “robotic surgery,” “gynecology,” “intuitive,” as well as various benign and malignant gynecologic conditions and surgical procedures. We also used a website named “All About Robotic Surgery” to review robotic surgery history (http://www.allaboutroboticsurgery.com/).

The review includes studies of various designs that include at least 25 women who had undergone robotic gynecologic surgery. Fifty-five studies (42 comparative and 13 non-comparative) met eligibility criteria.

## ROBOTIC SURGERY FOR BENIGN INDICATIONS

### Myomectomy

Four studies compared robotic myomectomy with either laparoscopic or open techniques. Two included open cohorts,^5^,^6^ two included laparoscopy,^6^,^7^ and one was a meta-analysis that compared all three techniques.^8^

Two studies summed up their experience in robotic myomectomy, one of which underlining the size of the myoma as the main focus of the study,^9^ and the other consisting of a more general overview of the surgery and its results.^10^

One retrospective study reported on the fertility outcome in a cohort of women treated by robotic myomectomy.^11^

Operative times were similar in the robotic and the laparoscopic myomectomy groups.^6^–^8^ Open myomectomy was much less time-consuming compared to robotic myomectomy (126–138.6 min versus 181–192.3 min, *P*<0.001).^5^,^6^

Regarding blood loss during surgery, Bedient et al.^7^ found a statistically significant advantage in favor of the robotic myomectomy group (100 mL versus 250 mL, *P*=0.02), an advantage that was not observed by Barakat et al.^6^ (100 mL versus 150 mL, *P*=0.08). In comparison to the open myomectomy group, estimated blood loss was significantly lower in the robotic myomectomy group (100–200 mL versus 100–437.5 mL).^6^

Length of post-surgical hospitalization was similar in the two groups according to Bedient et al.^7^ When compared to open myomectomy, Ascher-Walsh et al.^5^ and Barakat et al.^6^ found that length of post-surgical hospitalization was shorter with robotic myomectomy (0.51 day versus 3.28 days, *P*<0.001; and 1 day versus 3 days, *P*<0.001, respectively).

The afore-mentioned meta-analysis^8^ included eight studies that compared robotic and laparoscopic myomectomy and nine studies that compared robotic and open myomectomy. In total, 2,027 patients were included. Robotic myomectomy proved to be significantly inferior regarding operative time (85 min in the open myomectomy group), but proved its superiority in terms of estimated blood loss (93 mL in the robotic myomectomy group), the need for transfusion (981 patients in the open myomectomy group), total complications (1,101 patients in the open myomectomy group), and length of post-surgical hospitalization (1.84 days/ patient in the robotic myomectomy group). Long-term outcomes including pain management, fertility, and pregnancy rates postoperatively in addition to recurrence rates still need to be studied.

Gunnalaet al.^9^ examined the feasibility of robotic myomectomy in patients with myomas larger than 9 cm, comparing them in a retrospective case–control manner with patients with myomas smaller than 9 cm. A statistically significant increase in operative time (130 min versus 92 min) and estimated blood loss (100 mLversus 25 mL) was found in the ≥9 cm group. A myoma larger than 15 cm was seen in 4.8% of the patients, and specimen weight was over 900 g in 5.3% of the patients, with little effect on clinical adverse outcomes. Patients in both groups were discharged on the day of surgery. The main conclusion was that myoma size should not be a factor taken into consideration when attempting robotic myomectomy. Also, patients undergoing robotic myomectomy may be discharged on the day of surgery.

Asmar et al.^10^ reported their experience with 36 robotic myomectomies performed at the Foch hospital in Paris. The median post-surgical hospitalization time was 3.29 days. Post-surgical anemia (hemoglobin measured 9.5–10.5 g/dL) due to excessive blood loss was detected in 19.5% of the patients, none of whom required blood transfusion. Post-surgical pregnancy rates were as high as 80% among women desiring pregnancy. No surgical site infection, resistant pain, or re-hospitalization for any reason was documented among the patients.

Sangha et al.^11^ conducted a seven-year retrospective study of 310 women who had undergone myomectomy, 40% of whom desired pregnancy. Of the women desiring pregnancy 40% conceived; 61% of those who conceived delivered a viable infant in their first pregnancy, 6% in their second pregnancy (2 after a miscarriage and 1 after an ectopic pregnancy). Ten percent of the women desiring pregnancy delivered a second viable infant. Surgical technique, patient age or race, number of uterine incisions, and endometrial cavity invasion had no effect on the occurrence or outcome of pregnancy. Thus, myomectomy performed to preserve fertility resulted in approximately 25% live births, independent of surgical technique.

In conclusion of this section, robotic myomectomy is superior to laparoscopic and open myomectomy in terms of morbidity rates, esthetic results, adhesions, recovery, surgical accessibility (when compared to laparoscopic and not open surgery), and quality of sutures (when compared to laparoscopic and not open surgery, though there are insufficient data regarding uterine rupture during sequential pregnancies). Robotic myomectomy has the same effect on fertility compared to laparoscopic and open myomectomy. Robotic myomectomy is extremely expensive compared to the other surgical approaches, a fact restraining its access. Ultimately, randomized clinical trials (RCTs) are urgently needed to support the influx of data regarding the advantages of robotic surgery.

### Hysterectomy for Benign Indications

Six studies compared robotic hysterectomy for benign indications with either laparoscopic, abdominal, or vaginal techniques; two studies included either open or vaginal techniques,^12^,^13^ and all of them included laparoscopy.^12^–^17^ Five of the comparative studies were retrospective,^12^–^14^,^16^,^17^ and one was a randomized clinical trial.^15^

Four studies compared robotic and laparoscopic hysterectomies regarding operative times and clinical outcome. Landeen et al.^12^ found that there was no difference in surgical time (117.2 min versus 118.3 min). Sarlos et al.^14^ found that robotic surgery lasted longer than laparoscopic surgery (108.9 min versus 82.9 min, *P*=0.05). Swenson et al.^17^ also found that robotic surgery had longer operative times (2.3 h versus 2.0 h, *P*<0.001), though the study also took into account uterine weights, that were apparently larger in the robotic group (178.9–186.3 g versus 160.5–190 g, *P*=0.007). In the study by Moawad et al.^16^ there was a bias in the form of a higher body mass index (BMI) (32.9±6.5 versus 30.4±7.1, *P*=0.012) and more frequent history of adnexal surgery (12.9% versus 4.2%, *P*=0.031) in the robotic surgery group, while the laparoscopic group had a more frequent history of salpingectomy (81% versus 66.3%, *P*=0.02). Interestingly, although maybe due to the bias, laparoscopic hysterectomies had longer operative time, adding 47 min (31–63 min, *P*=0.001).

Swenson et al.^17^ found that the rate of post-surgical complications was lower in the robotic surgery group (3.5% versus 5.6%, *P*=0.01), including lower rates of surgical site infection (0.07% versus 0.7%, *P*=0.01) and need for blood transfusion (0.8% versus 1.9%, *P*=0.02). Major post-surgical complications such as intraoperative bowel and bladder injury, readmissions, and the need for reoperations were similar between groups. Thus, robotic hysterectomy did not decrease major morbidity following hysterectomy for benign indications when compared to laparoscopic hysterectomy. Though total complications were lower, in the absence of substantial reductions in clinically and financially burdensome complications, it seems that hysterectomy for benign indications via robotic technique is not clinically superior or cost-effective.

Estimated blood loss and length of post-surgical hospitalization were reduced with robotic hysterectomy in three out of four studies (*P*<0.0001). Only Moawad et al.^16^ found that estimated blood loss was the same with both techniques.

Two retrospective studies conducted by Landeen et al.^12^ and Matthews et al.^13^ compared robotic and open hysterectomy. Landeen et al.^12^ found that robotic hysterectomy required longer operative time (117.2 min versus 83.7 min, *P*<0.001). Both studies found in the robotic hysterectomy a 2- to 4-fold reduction in estimated blood loss (82.3 mL versus 430 mL, *P*<0.001; and 109.3 mL versus 269.8 mL, *P*=0.001), and a 50% reduction in length of post-surgical hospitalization (1.5 days versus 3.5 days, *P*=0.001; and 1.3 days versus 2.7 days, *P*<0.001).

The only RCT comparing robotic versus laparoscopic hysterectomy for benign indications so far was conducted by Deimling et al.^15^ Seventy-two patients were randomized to each surgical arm. Mean operative time was the same in both surgical groups: 73.9 min (median 67.0 min; interquartile range 59.0–83.0 min) in the robotic hysterectomy group, and 74.9 min (median 65.5 min; interquartile range 57.0–90.5 min) in the laparoscopic hysterectomy group. The study concluded that when performed by a surgeon experienced in the chosen technique, robotic hysterectomy was non-inferior, in terms of operative time, to laparoscopic hysterectomy.

### Sacrocolpopexy

Six studies compared robotic sacrocolpopexy (SCX) to either laparoscopic,^18^–^20^ abdominal,^20^,^21^ or vaginal^22^ approaches; two studies were RCTs,^18^,^22^ and two were cost-effectiveness tests.^20^,^23^

Two case series concerned robotic SCX, one of which summarized cases regarding robotic SCX for the treatment of vaginal vault prolapse,^24^ the other reporting on cases regarding a new approach in robotic SCX, the single-port approach.^25^

One study evaluated the impact of obesity on robotic SCX.^26^

Paraiso et al.^18^ conducted a RCT in which 35 women had undergone robotic SCX and 33 women had undergone laparoscopic SCX. In the laparoscopic SCX group, total operating room time was shorter (199 min versus 265 min, *P*<0.001): shorter SCX time (162 min versus 227 min, *P*<0.001) and shorter SCX suturing time (68 min versus 98 min, *P*<0.001). Post-surgical hospitalization was similar in the two groups (43 h versus 34 h, *P*=0.17).

Two studies^19^,^21^ retrospectively compared laparoscopic and robotic SCX. Estimated blood loss was lower in the robotic SCX group in both studies (*P*<0.0001). Awad et al.^19^ found that mean operative times did not differ significantly (176 min [110–380] versus 186 min [105–345], *P*=0.34); however, Geller et al.^21^ found that robotic SCX had longer operative times (328 min versus 105 min, *P*<0.001). In both studies, mean post-surgical hospitalization was shorter for the robotic group (*P*<0.0001). There were no significant adverse events in either group.

Two cost-effectiveness studies^20^,^23^ were performed to compare robotic and open SCX. Judd et al.^20^ compared robotic, laparoscopic, and open SCX approaches and found that the robotic approach was the most expensive one. Elliott et al.^23^ found that open SCX was less expensive than robotic SCX, possibly due to differences such as inclusion of hysterectomy, longer operating time, and higher cost of disposable instruments.

Westermann et al.^22^ conducted a RCT that compared perioperative pain and recovery on post-operative day 1, and at 2 and 6 weeks post-surgery, between women who had undergone vaginal hysterectomy with uterosacral ligament suspension (USLS) and robotic SCX. Each group included 39 women. In the robotic SCX group patients had lower nursing verbal pain scores (*P*=0.04), less narcotic consumption (*P*=0.02), and lower estimated blood loss (*P*=0.01). Operating time was longer in the robotic group (*P*<0.001). At 2 and 6 weeks post-surgery, there were no significant differences between the two groups. The investigators concluded that both approaches had similar quality-of-life scores after surgery. The robotic approach is associated with less pain and less narcotic use post-surgery.

Pellegrino et al.^24^ retrospectively evaluated the feasibility and clinical outcomes of robotic SCX for the treatment of vaginal vault prolapse in 31 consecutive cases. Mean follow-up time was 27 months (range 2–48). Average total operative time was 185 min (range 170–235). Estimated blood loss was 50 mL (range 30–150). Except for one case of cystotomy, no other intraoperative complications occurred. Successful outcome was reported in 94% of the patients.

Kissane et al.^25^ retrospectively compared operative times of robotic SCX across a range of BMI values. They compared 179 women, 61 (34%) of whom were normal weight (BMI 25 kg/m^2^), 72 (40%) of whom were overweight (BMI 25–30 kg/m^2^), and 46 (26%) of whom were obese (BMI 30+ kg/m^2^). Overweight patients were significantly older, more parous, more frequently postmenopausal, and more frequently had undergone concomitant salpingo-oophorectomy. Median operative time was 202, 206, and 216 min, respectively (*P*=0.53).

### Robotic Single-port SCX

Matanes et al.^26^ reviewed a state-of-the-art robotic surgical technique, single-port SCX, during which the surgeon operated almost exclusively through a single entry point, leaving only a single small scar. The investigators’ aims were to evaluate the new technique’s learning curve and, in addition, to share tips for improved single-port robotic SCX based on the first 25 patients to have undergone single-port robotic SCX. Median age was 59 years (range 35–74). Median “pelvic organ prolapse quantification” stage was 3 (range 2–4). Median total operative time was 190 min (range 114–308). Median console time was 130 min (range 85–261). A comparison between the first 15 cases and the next 10 cases demonstrated significant reductions in median operative times and console times: 226 min (range 142–308) versus 156 min (range 114–180), and 170 min (range, 85–261) versus 115 min (range 90–270), respectively (*P*<0.008). No intraoperative adverse events occurred in any of the cases. Postoperative adverse events were extremely rare and included one case of small-bowel adhesions that required a second laparoscopic surgery for adhesiolysis and led to the addition of mesh peritonization in all the successive cases; median peritonization time was 8 min (range 5–15 min). The investigators concluded that single-port robotic SCX was a feasible technique with lower complication rates, minimal blood loss and postsurgical pain, faster recovery, shorter post-surgical hospitalization, and virtually scar-free results.

### Endometriosis

Chen et al.^27^ conducted a meta-analysis meant to evaluate the safety and efficacy of robotic versus laparoscopic surgery for the treatment of advanced-stage endometriosis. Due to lack of suitable clinical trials only two studies were included. No significant differences were observed between the two groups in terms of estimated blood loss, complication rate, and post-surgical hospitalization. Mean operative time in the robotic surgery group was longer (73.85 min, *P*<0.00001). Thus, the benefits of robotic surgery over laparoscopic surgery in the treatment of advanced-stage endometriosis remain uncertain.

### Conversion from Robotic Approach to Other Approaches

Unger et al.^28^ attempted retrospectively to determine the incidence of, and risk factors for, conversion from robotic procedures to other surgical techniques. Patients’ demographic and perioperative data were retrieved, in addition to surgeon experience based upon monthly case volume. During the period reviewed 942 robotic procedures were performed. Conversion from robotic to any other surgical technique was recorded for 47 procedures (5.0%), of which 16 (1.7%) were conversions to open surgery. Conversion from robotic surgery to another surgical technique was associated with higher BMI (*P*=0.001), previous laparotomy (*P*=0.042), and poor surgeon experience (*P*=0.011). Asthma (*P*=0.008), intraoperative bowel injury (*P*<0.001), intraoperative vascular injury (*P*=0.003), and single-port robotic surgery (*P*=0.034) were associated with increased odds for conversion.

## ROBOTIC SURGERY FOR MALIGNANT INDICATIONS

### Robotic Surgery for Treatment of Endometrial Cancer

Twenty-one studies compared robotic surgery for endometrial cancer with either laparoscopic or abdominal approaches; 14 studies included a comparison to laparoscopic approaches,^29^–^42^ while 11 studies included a comparison to open approaches,^30^,^31^,^34^,^36^,^43^–^49^ and one was a randomized clinical trial.^42^

One study was a prospective study^50^ regarding single-site approach for endometrial cancer staging.

### Robotic Versus Laparoscopic Surgery

Fourteen observational studies assessed operative time and length of post-surgical hospitalization comparing robotic and laparoscopic surgery for endometrial cancer.^29^–^42^ Length of post-surgical hospitalization was reduced in the robotic groups. There was some inconsistency in the finding of shorter operative times in the laparoscopic surgery groups. The largest study reporting operative times was by Barrie et al.^40^ (*n*=1,433), pointing to shorter operative times in the robotic surgery group whether the procedure included hysterectomy alone or addition of pelvic/para-aortic lymph node dissection. Statistically significantly shorter times were observed in the patients undergoing hysterectomy alone (125 min [108–151] versus 136 min [111–171], *P*=0.02) and the patients undergoing hysterectomy, pelvic lymph node dissection, and para-aortic lymph node dissection (186 min [154–232] versus 244 min [205–279], *P*<0.01). These results were supported by the only RCT conducted in this context until now, performed by Mäenpää et al.,^42^ in which 99 patients were randomly assigned to two groups: robotic and laparoscopic surgery. Operative times in the robotic surgery group were shorter (139 min [range 86–197] versus 170 min [range 126–259], *P*<0.001). Furthermore, there were no differences in the number of lymph nodes removed, estimated blood loss, and length of post-surgical hospitalization between the two groups. In the laparoscopic surgery group five conversions to open surgery occurred as opposed to zero conversions in the robotic surgery group (*P*=0.027). There were more major postoperative complications in the robotic surgery group (11 versus 5, *P*=0.111). Thus, Mäenpää et al.^42^ concluded that robotic surgery offers an effective and safe alternative in the surgical treatment of endometrial cancer.

The majority of studies found that estimated blood loss was significantly lower in the robotic surgery groups.

When comparing robotic surgery with laparoscopic surgery regarding the total number of lymph nodes removed, four studies reported robotic superiority, three studies reported robotic inferiority, and four studies showed no difference.

### Robotic Versus Open Surgery

Eleven studies compared robotic and open surgery for the treatment/staging of endometrial cancer.^30^,^31^,^34^,^36^,^43^–^49^ Robotic surgery was by far superior to open surgery regarding estimated blood loss and post-surgical hospitalization. Except for two studies (one by El Sahwi et al.^45^ that showed shorter operative times for the robotic surgery group, and the other by Hinshaw et al.^49^ that showed similar operative times in both surgical groups), all found operative times to be longer in the robotic surgery groups. The total number of lymph nodes retrieved (which is considered a surgery quality indicator) was different in both groups, with five studies reporting robotic superiority, two studies reporting robotic inferiority, and five studies reporting no difference.

From an economic aspect, robotic surgery has a reputation of being costly compared to other surgical approaches because of the high cost of robotic surgical sets and disposable parts, but after stratification and review of costs of extended post-surgical hospitalization in the open surgery groups compared to the robotic surgery groups, it is inferred^30^,^47^ that robotic surgery is less costly than open surgery (*P*<0.001).

### Robotic Single-port Surgery for Endometrial Cancer

Corrado et al.^50^ evaluated the feasibility and safety of robotic single-site hysterectomy either with or without pelvic lymph node dissection. The investigators prospectively collected clinical and operative data, as well as data on length of stay, on all patients who had undergone the afore-mentioned surgery for clinical International Federation of Gynecology and Obstetrics (FIGO) stage I or occult stage II endometrial carcinoma. A total of 125 patients were included in the study. Median docking, console, and total operative times were 11 min (range 4–40 min), 80 min (range 20–240 min), and 122 min (range 35–282 min), respectively. Median estimated blood loss was 50 mL (range 10–250 mL). The only conversion to a different surgical approach was in one patient who was converted to vaginal surgery due to the patient’s pulmonary baseline morbidity. Pelvic lymphadenectomy was performed in 16.8% of the patients, while the median number of lymph nodes retrieved was 13 (range 3–32). Median post-surgical hospitalization was 2 days (range 1–3 days). No intraoperative complications were documented. The investigators concluded that robotic surgery is technically feasible, safe, and reproducible for this indication and grade of disease, and has the potential to become the treatment of choice for patients affected by FIGO stage I–II endometrial cancer. However, randomized controlled trials are needed to confirm these results.

### Robotic Surgery for Treatment of Cervical Cancer

A total of seven studies compared robotic radical hysterectomy with either laparoscopic hysterectomy^51^ or open radical hysterectomy.^52^–^57^ In addition, we chose to include two more studies: one summary of 3 years of robotic radical hysterectomy experience^58^ and one prospective non-randomized phase II study.^59^

Soliman et al.^51^ conducted the only study that compared laparoscopic and robotic radical hysterectomy approaches. The investigators found that both approaches showed similar operative times, length of post-surgical hospitalization, and total number of lymph nodes retrieved. Estimated blood loss was significantly lower in the robotic surgery group (115.5 mL versus 171 mL, *P*<0.001).

Seven studies evaluated and compared operative times, length of post-surgical hospitalization, and estimated blood loss between robotic and open radical hysterectomy.^52^–^57^ All of the studies found similar results regarding significantly shorter length of post-surgical hospitalization after robotic surgery, ranging from 1 to 3.7 days for robotic-assisted procedures and 2.8 to 5 days for open surgery.

Nam et al.^55^ reported longer hospitalization periods, nearly 3-fold the reported average (robotic, 11.6 days; open, 16.9 days), most likely due to different practice patterns. All seven studies reported significantly lower estimated blood loss, with decreases of 49% to 77% in the robotic surgery groups. Some inconsistencies were found between the different studies regarding operative times. Two studies^54^,^55^ found no significant differences between the two surgical approaches. Three studies^51^,^53^,^57^ found that robotic surgery required longer operative times (*P*<0.001). The two remaining studies^52^,^58^ showed the exact opposite results regarding operative times, reporting that robotic surgery had shorter operative times (*P*=0.002). These inconsistencies are probably but not solely the result of surgeon’s experience.

Cantrell et al.^58^ evaluated the 3-year survival of patients who had undergone radical hysterectomy, whether robotic, laparoscopic, or open. No difference in overall survival was observed between the different groups. Recurrence was rare and similar between groups. Pelvic lymph nodes were dissected in 98% of patients, and were found to be positive for disease in 8.5%–10.9% of patients. The mean number of pelvic lymph nodes retrieved was higher in the minimally invasive group (19.4 versus 16.0, *P*<0.001). There was no difference in the rate of post-operative chemotherapy (*P*=0.32) or radiation therapy (*P*=0.28).

Gallotta et al.^59^ conducted a prospective non-randomized controlled trial (Canadian Task Force classification level 2) enrolling patients with stage IB2–III cervical cancer who underwent robotic radical hysterectomy plus pelvic and/or aortic lymph node dissection within 6 weeks after chemotherapy/radiation therapy. Surgery feasibility and complications were analyzed. Pelvic lymph node dissection was performed in all cases. Robotic surgery was successful in 97.5% of cases. Median operative time was 185 min (range 100–330 min), and median estimated blood loss was 100 mL (range 50–300 mL). Median length of post-surgical hospitalization was 2 days (range 1–4 days). No intraoperative complications were recorded. During the observation period, 30% of the patients had complications. Recurrence was documented in 12.5% of patients.

### Robotic Surgery for Staging of Ovarian Cancer

The one study published to date comparing robotic and laparoscopic approaches found no statistically significant difference between the two approaches with regard to final FIGO stage, histology, and tumor grade.^60^ In addition, 15.6% of the patients were upstaged, with no statistically significant difference between the two groups. Median number of pelvic lymph nodes retrieved was 14 (range 3–42) and 11 (range 2–29) in the robotic and the laparoscopic groups (*P*=0.235), respectively. Median number of aortic lymph nodes retrieved was 11 (range 3–26) and 12 (range 1–39) in the robotic and the laparoscopic groups (*P*=0.263), respectively. Operative time was significantly shorter in the robotic group (*P*=0.043). Estimated blood loss was similar (*P*=0.691). No difference was found in terms of surgical complications.

## DISCUSSION

The experience of the last 12 years has been documented meticulously in the various studies reviewed above. The evolution of robotic surgery is quite phenomenal for such a young technology, perhaps in part due to the obvious advantages of robotic surgery in general. The majority of studies available today regarding robotic surgery are retrospective and based on a single surgeon’s/center’s experience. The number of RCTs available is extremely limited, thus making it difficult for administrators, reviewers, or health care givers to assimilate and create clear clinical guidelines regarding the use of this new technology.

The majority of studies available suggest that in most benign indications the robotic approach is non-inferior or superior to the laparoscopic approach and consistently proves to be superior to the open approach. When it comes to malignant indications, the results are quite similar, though robotic surgery for the treatment of endometrial cancer has been receiving excellent reviews in the field and in clinical studies.

Most of the reviewed studies compared three main aspects of robotic surgery including operative time, estimated blood loss, and length of post-surgical hospitalization.

Robotic surgery was by far superior to both abdominal and laparoscopic approaches regarding both estimated blood loss and length of post-surgical hospitalization. On the other hand, the results regarding operative times were inconsistent, most probably due to the variance in surgeon experience, likely a factor in the high cost of surgery. The learning curve described in the reviewed studies points to between 20 and 30 surgeries being required to begin mastering the robotic technique. In the investigators’ experience, previous experience in laparoscopic surgery is a relative advantage before embarking on the robotic surgery adventure. Previous gaming (video-game) experience may also be a relative advantage in this context.

This review has several limitations, most of which result from the quality of evidence-based medicine available regarding robotic surgery in gynecology. Indicators of poor-quality studies include factors such as small number of patients included, generalization of robotic procedures in the same study due to low surgery volume, and very few RCTs.

This review is the most comprehensive and most up-to-date review available today regarding robotic surgery. That being said, RCTs regarding comparisons between different minimally invasive surgery techniques are required. Since cost-effectiveness is the greatest limitation of robotic surgery, future studies must include cost evaluations including calculations regarding length of hospitalization, post-surgical complications, and return to regular routine and recuperation.

Based on current knowledge and in light of the data reported in this review, in the case of gynecological surgery, the choice regarding surgical approach should be individualized based on patient background, surgeon’s experience, and availability of robotic instrumentation. When the time comes and robotic surgery is as common as laparoscopic surgery, there is no doubt that the abdominal approach will be abandoned, although, like with other important skills, surgeons will have to master open surgery before mastering minimally invasive surgery techniques, just in case of occurrence of complications or clinical situations requiring conversion to open surgery.

When reviewing the literature, we found quite a few case reports that described unique surgeries performed in a variety of gynecological indications, including ovarian indications requiring surgery,^60^ and the next step in robotic surgery aims at surgical site minimization—single-port robotic surgery.^26^,^61^,^62^ As proved by these publications, robotic surgery is still evolving, and the future is wide and unknown.

Currently (according to ClinicalTrials.gov) there are 11 RCTs being conducted worldwide, eight regarding robotic sacrocolpopexy or robotic pelvic organ prolapse repair (including NCT01535833, NCT02367235, NCT01346436, NCT03034499, NCT02676973, NCT02741830, NCT02852512, NCT02800512), four of which are comparative studies meant to compare robotic and either laparoscopic or vaginal approaches. One study regarding robotic uterine transplantation (NCT02987023) is being conducted. Two oncological studies are being conducted utilizing robotic surgery, one regarding cervical cancer, a comparative study meant to compare robotic and other minimally invasive approaches for the treatment cervical cancer (NCT00614211), and the other meant to evaluate the use of robotic surgery in gynecologic oncology (NCT00671827).

The data from the afore-mentioned studies will help evaluate the role of robotic surgery in gynecology and forge the future of robotic surgery in the field of gynecology.

## Abbreviations

FDA: Food and Drug Administration
FIGO: International Federation of Gynecology and Obstetrics
MASH: mobile advanced surgical hospital
RCT: randomized clinical trial
SCX: sacrocolpopexy
